# Supplementary material for: ENO1 Regulates Apoptosis Induced by Acute Cold Stress in Bovine Mammary Epithelial Cells
Source: Animals (Basel). 2025 Aug 31;15(17):2559. doi: 10.3390/ani15172559 (PMC12427403; doi:10.3390/ani15172559)
Supplement: Supplementary file 1 [file animals-15-02559-s001.zip › animals-3789148-supplementary.pdf]

Figure3 B.WB original image

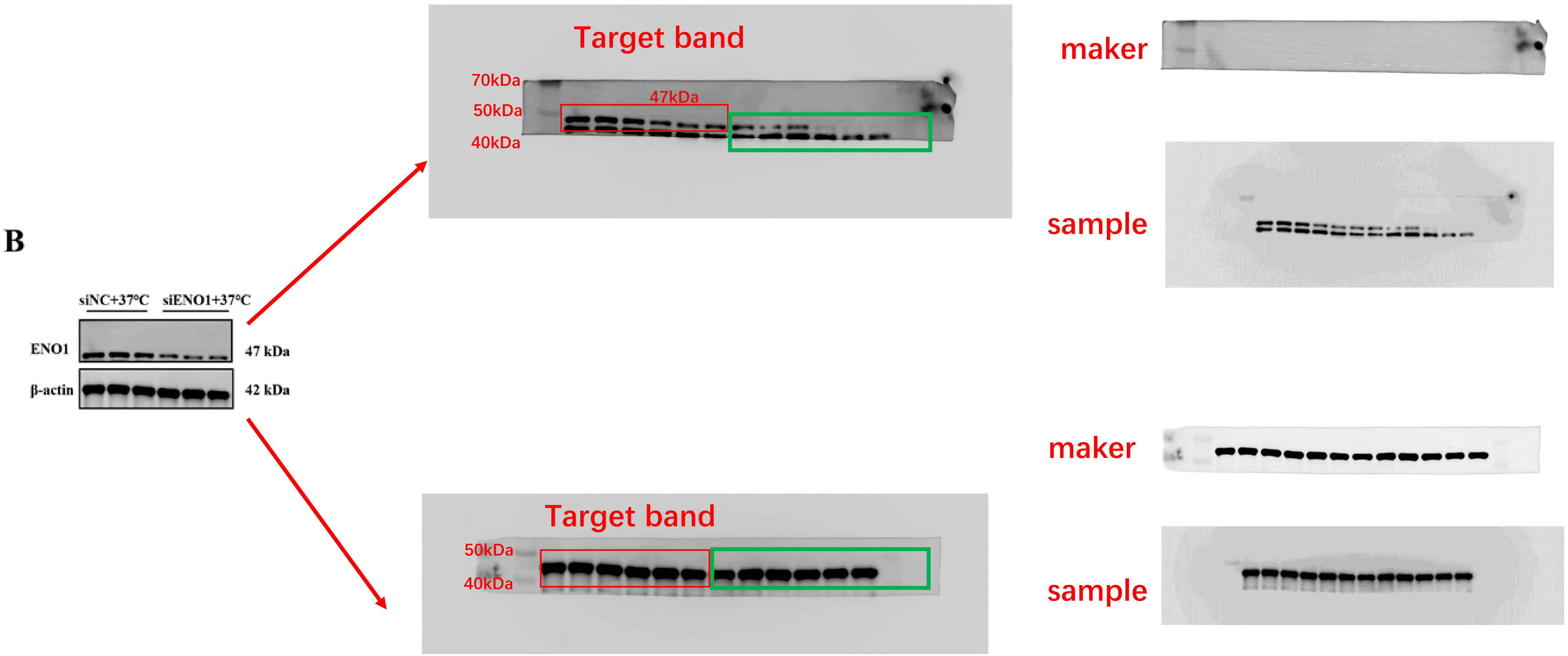

Note: The strips within the green box represent samples from the same period that were not included in the current study's analysis.

Figure3 B.WB original image

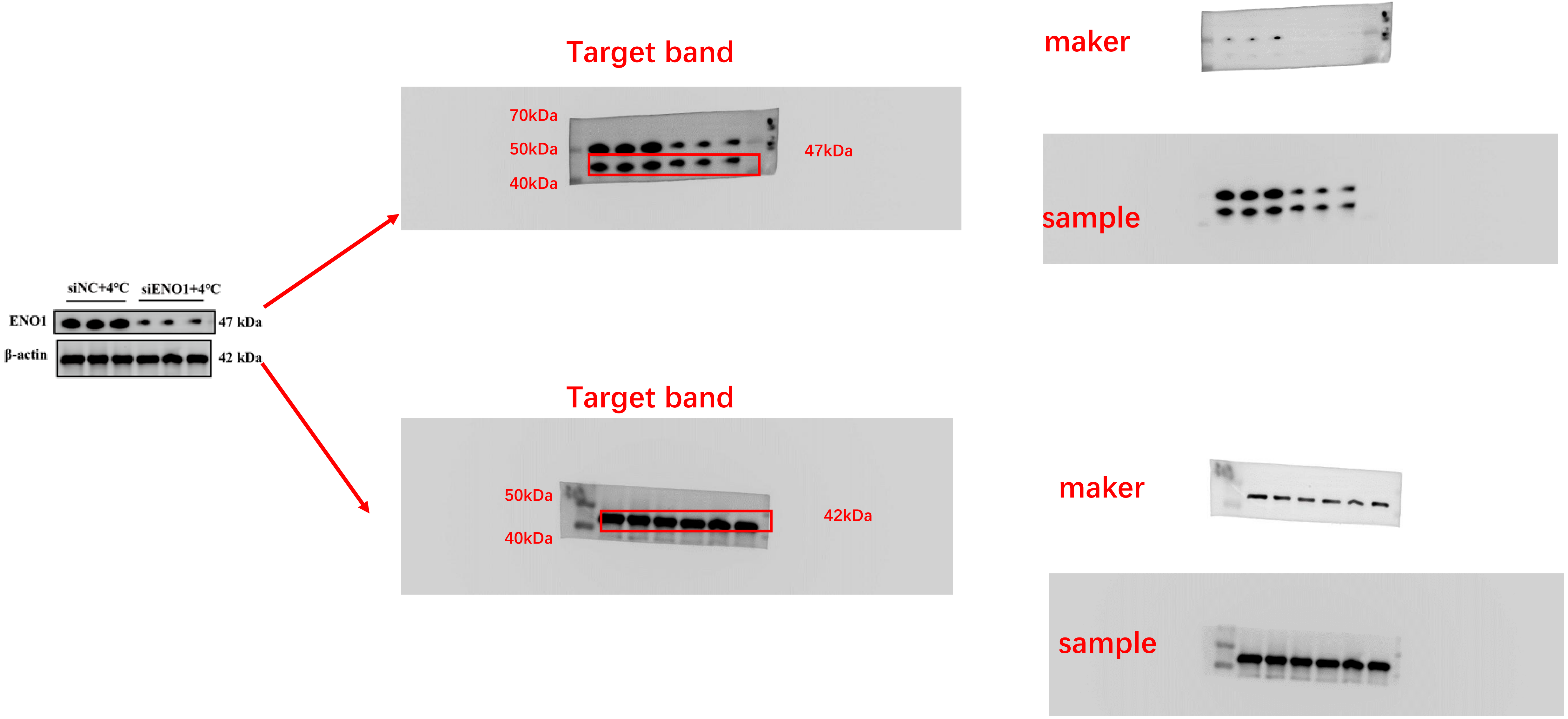

Figure3 H.WB original image of HSP90

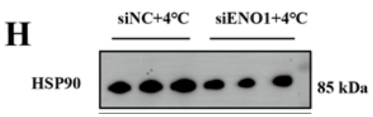

Target band

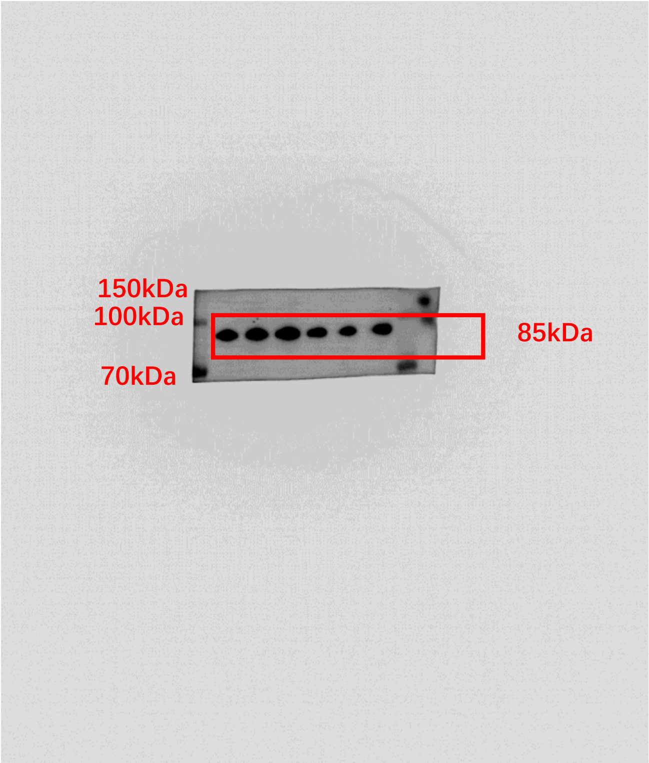

maker

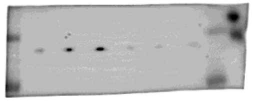

sample

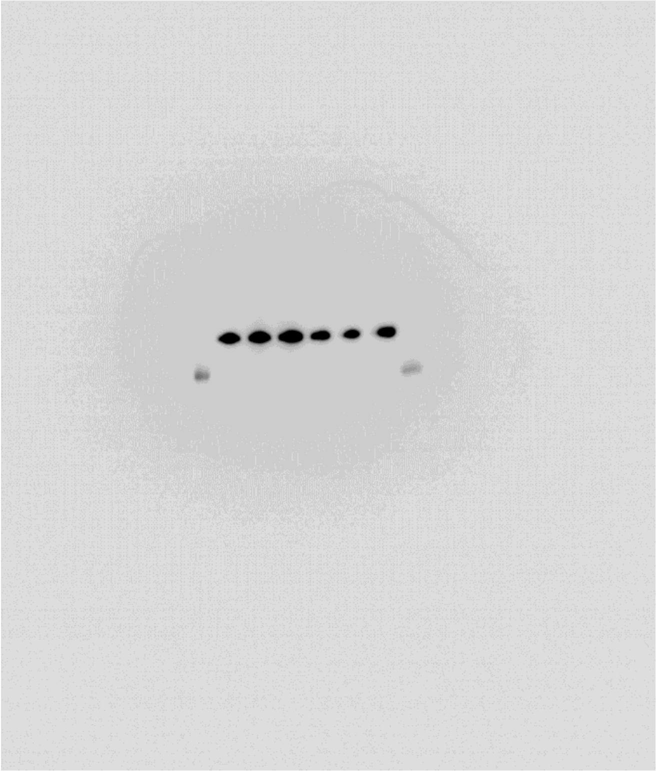

Figure3 H.WB original image of ENO1

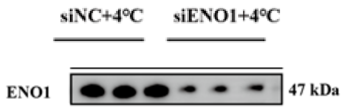

Target band

maker

sample

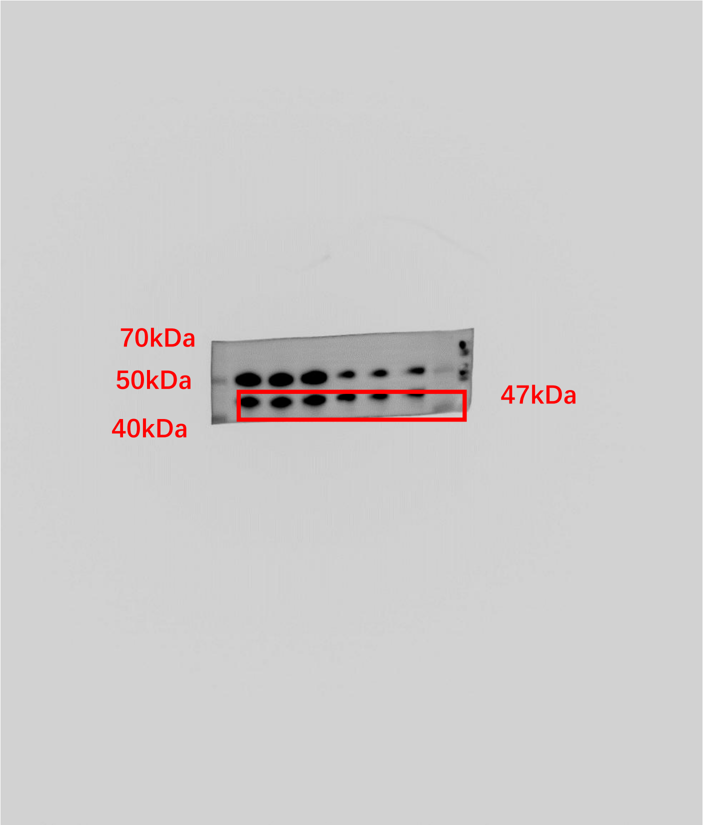

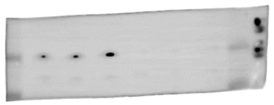

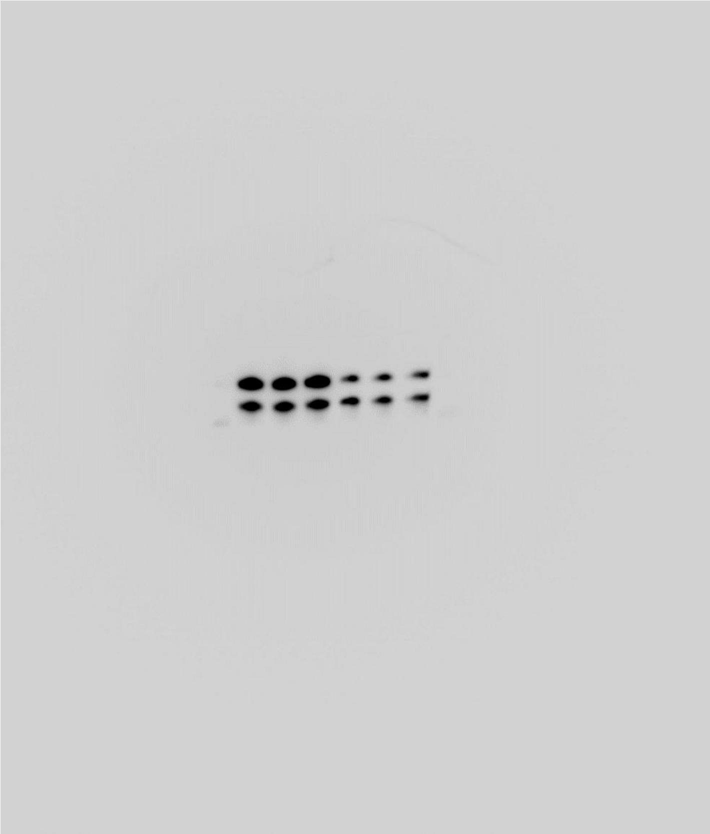

Figure3 H.WB original image of BCL-2

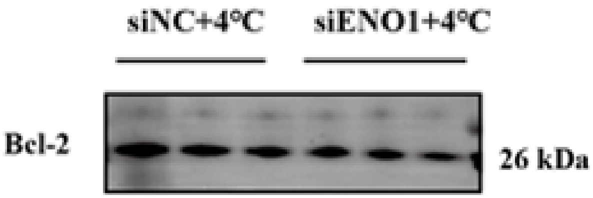

Target band

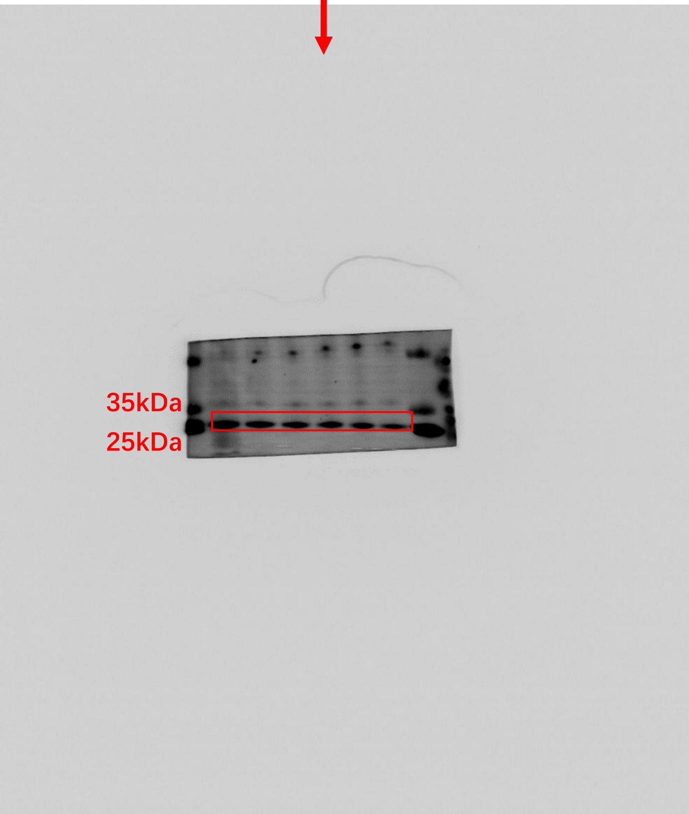

maker

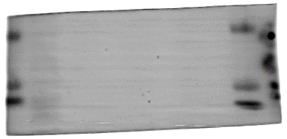

sample

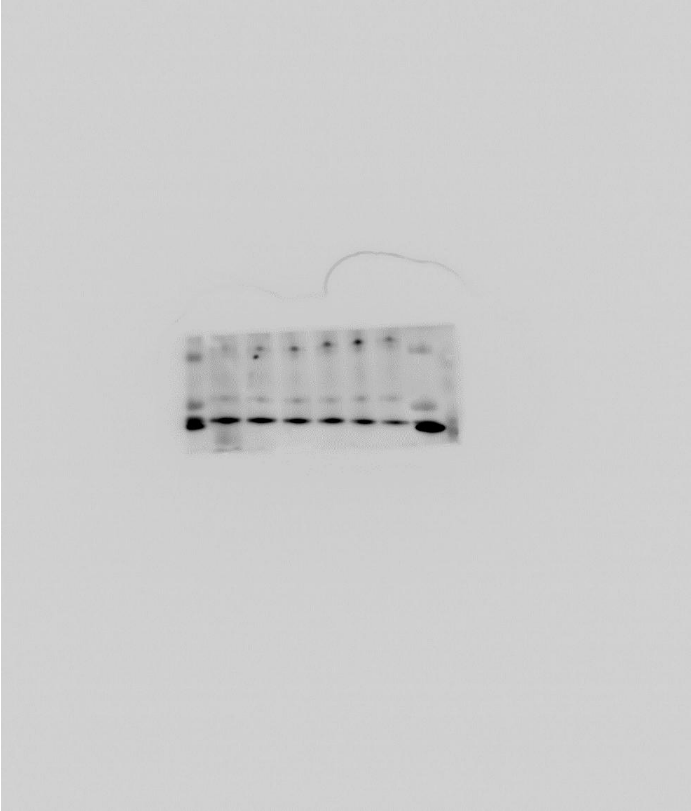

Figure3 H.WB original image of actin

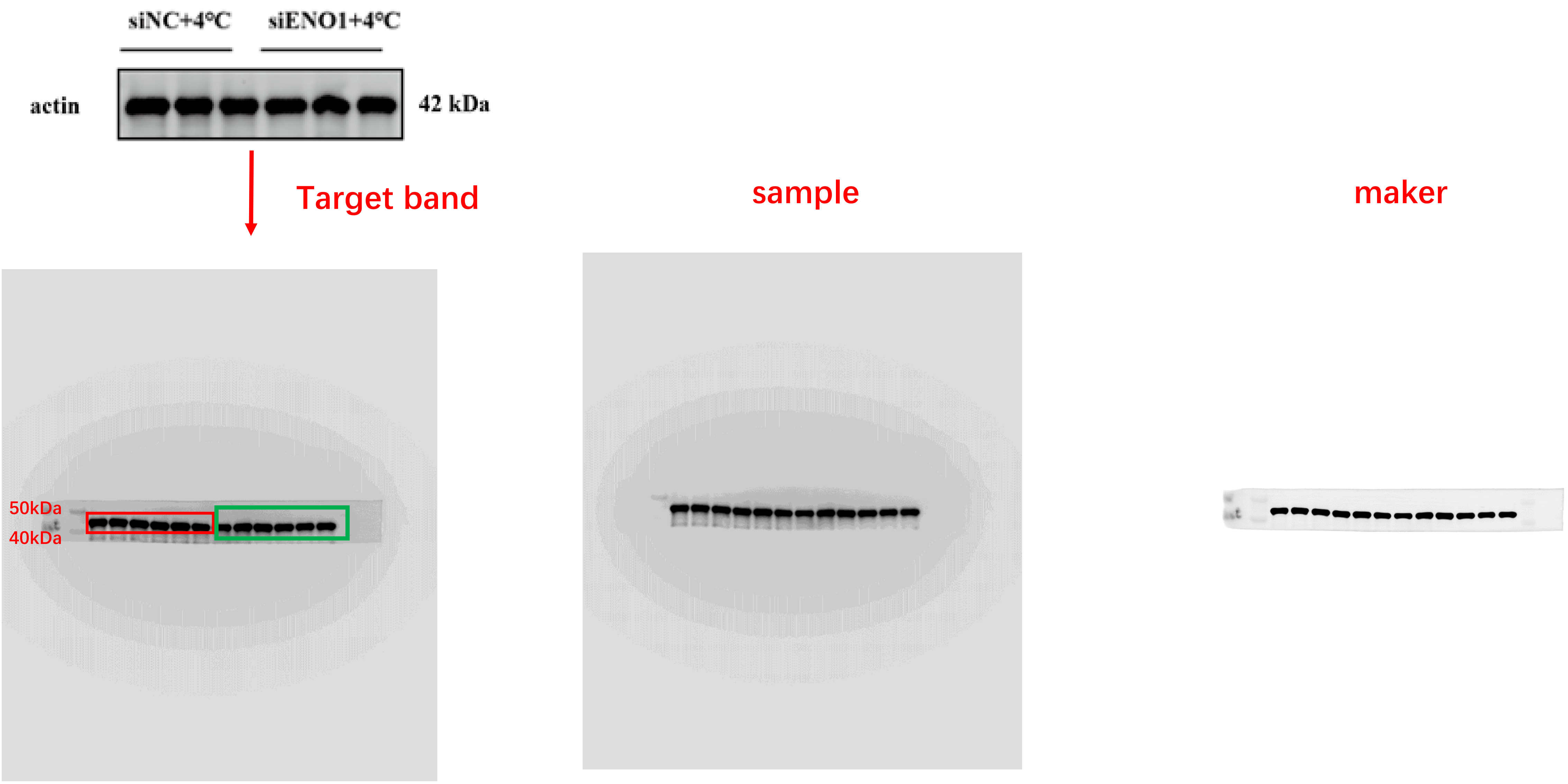

Note: The strips within the green box represent samples from the same period that were not included in the current study's analysis.

Figure3 H.WB original image of Casp3

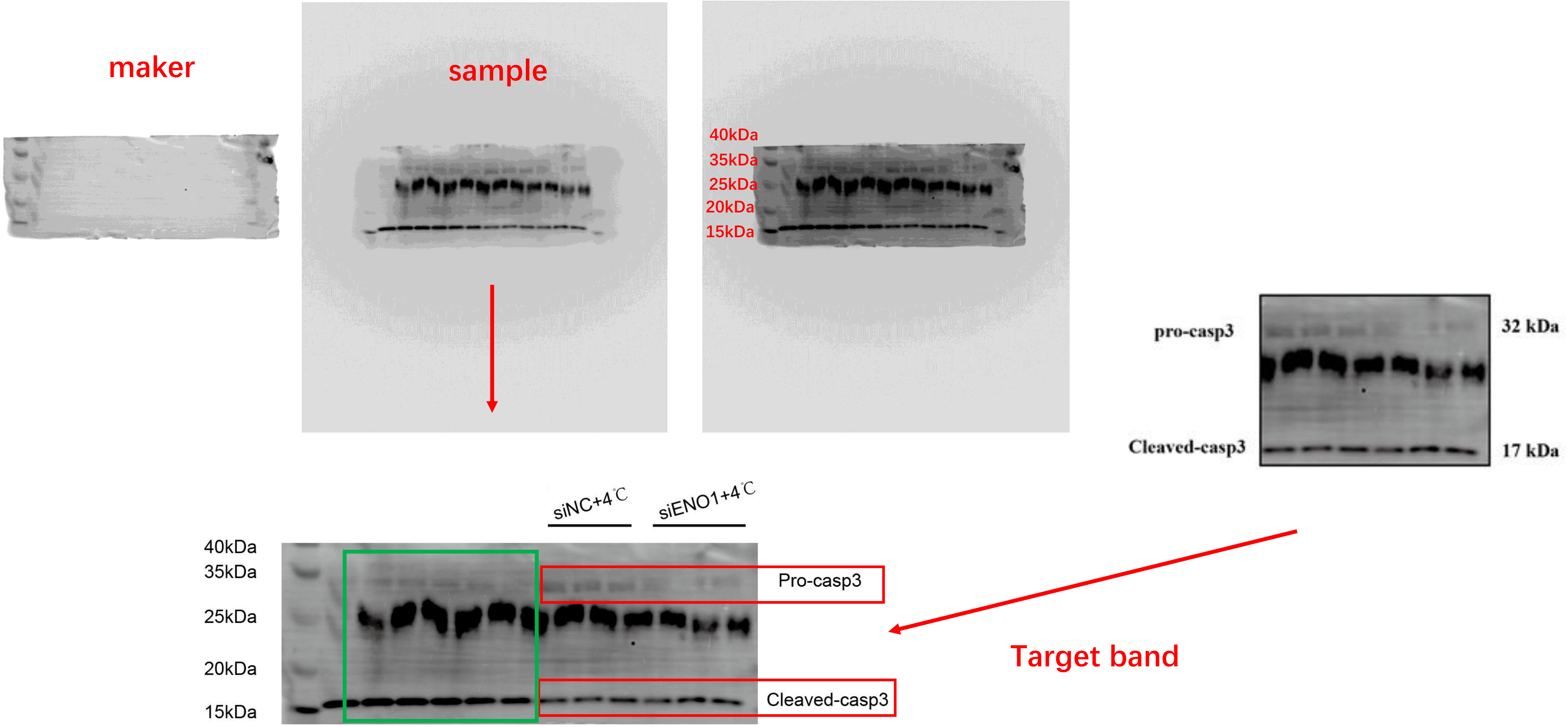

Note: The strips within the green box represent samples from the same period that were not included in the current study's analysis.

Figure3 H.WB original image Bax

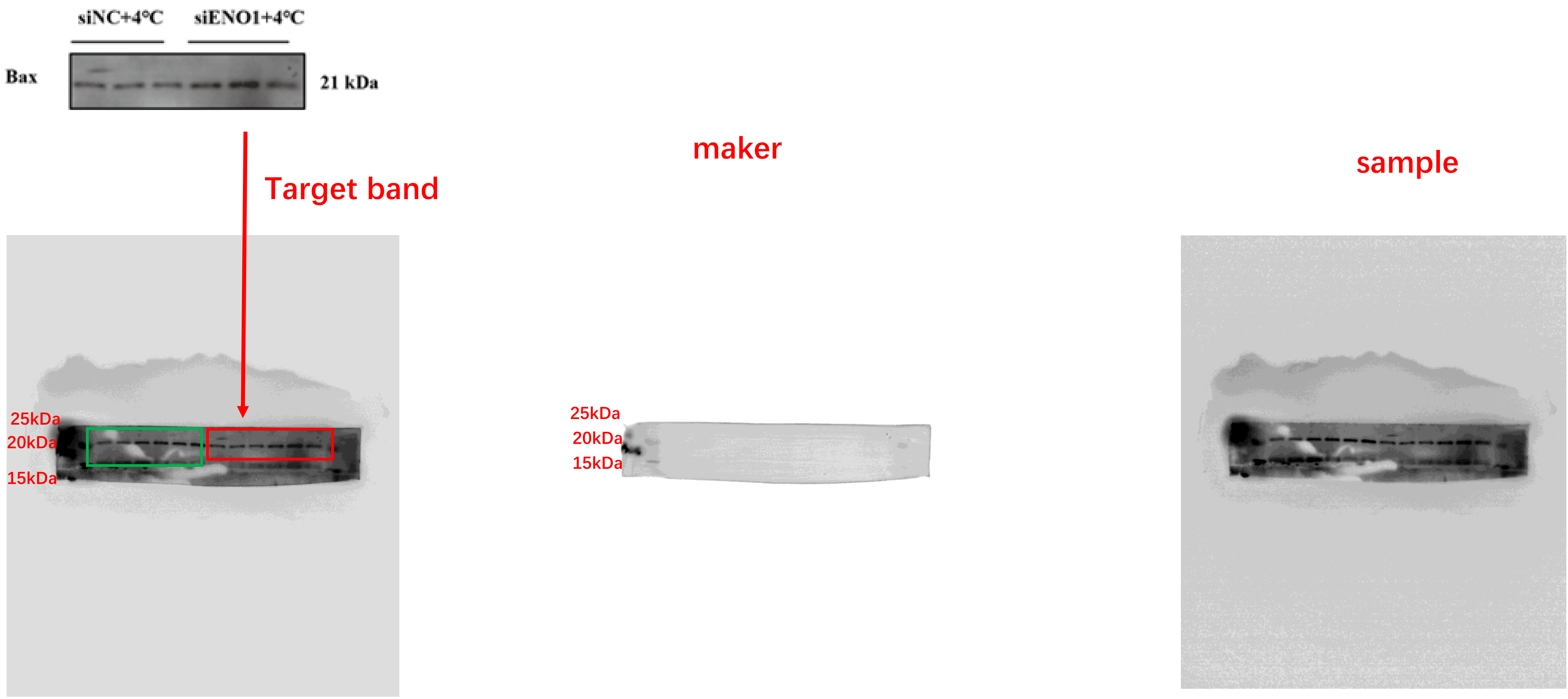

Note: The strips within the green box represent samples from the same period that were not included in the current study's analysis.
